# Contraceptive nonuse among women in Uganda: a comparative assessment of predictors across regions

**DOI:** 10.1186/s12905-020-01148-6

**Published:** 2020-12-17

**Authors:** Jude Otim

**Affiliations:** Department of Sociology and Social Administration, Kyambogo University, P.O. Box 1, Kyambogo, Kampala, Uganda

**Keywords:** Contraceptive nonuse, Regional, Fertility, Women, Uganda

## Abstract

**Background:**

Contraceptive nonuse has diverse effects on women, such as unintended pregnancies and births that result in high fertility and poor maternal health outcomes. In Uganda, knowledge on contraceptive use is high, amidst undesirably high contraceptive nonuse and scarce literature on predictors of contraceptive nonuse across regions. This study assessed factors associated with contraceptive nonuse among women of reproductive age across regions in Uganda.

**Method:**

This study used data from a cross-sectional 2016 Uganda demographic and heath survey that had 18,506 women of reproductive age. The relationship between contraceptive nonuse and socio-economic and demographic factors across regions were assessed using a binary multivariable logistic regression model.

**Results:**

In Uganda, contraceptive nonuse is estimated at 40%. Northern region (55%) had the highest prevalence of contraceptive nonuse compared to Central region (35%) with the lowest. Across regions, wealth index, number of living children, educational level, and children born in the last 5 years prior to the demographic survey differently predicted contraceptive nonuse. Conversely, age, religion, age at first marriage, sexual autonomy, age at first birth, desire for children, listening to radio, and employment status were only predictors of contraceptive nonuse in particular regions amidst variations. Residence, perception of distance to health facility, watching television, and reading newspapers or magazines did not predict contraceptive nonuse.

**Conclusions:**

The study findings propose the need to appreciate regional-variations in effect of contraceptive nonuse predictors and therefore, efforts should be directed towards addressing regional-variations so as to attain high contraceptive usage across regions, and thus reduce on unwanted pregnancies and births.

## Background

Worldwide, an estimated 190 million women (15–49 years) do not use contraceptives; most of whom are found in sub-Saharan Africa (83%) [1, 2]. Contraceptive nonuse among women of reproductive age in sub-Saharan Africa accounts for nearly 14 million unplanned pregnancies annually and majority of maternal deaths (66%) [3–7], amidst geographical variations [8, 9]. Uganda continues to present undesirable fertility rates (5.4 births per woman) and maternal mortality ratios (336 maternal deaths per 100,000 live births) [10, 11], that are associated with contraceptive nonuse [12–17]. In Uganda, almost everybody (99%) has knowledge on contraceptive use [10]. However, the knowledge is not equitable to current contraceptive uptake (39%) [10]; attributed to indistinct factors, particularly across regions of the country.

Studies on contraceptive nonuse in Uganda report socio-economic and demographic preditors such as; educational level, age, wealth status, fear of side effects, residence, low quality of contraceptive services, alcohol intake, income, sex, and age at first sex [16, 18–20], without examining predictors’ across regions. Conspicuously, wide variations in contraceptive nonuse, and consequent fertility, and maternal mortality exist within regions in Uganda despite continued good strategies and rigorous efforts to lower contraceptive nonuse in the country [10, 21]. For instance; Karamoja region has the highest prevalence of contraceptive nonuse (92.7%) among currently married women (15–49 years), whilst highest fertility (7.9 children per woman) and maternal mortality ratio (588 per 100,000 live births), compared to other regions [10]. Therefore, differences in contraceptive nonuse across regions suggest regional-specific predictors of the vice.

Uganda is composed of four administrative regions, and they include; Eastern, Northern, Central, and Western regions [22]. These regions have variations in livelihood sources, as well as levels of poverty [22]. For instance; Eastern region is known to be the poorest of all regions in the country [23]. In addition, Karamoja region is reportedly the least social and economically developed region [24]. Studies indicate that discrepancies in poverty and sources of livelihood have a bearing on contraceptive nonuse [23, 25, 26]. Therefore, this study underscores the need to seek address of this gap through examining the association between contraceptive nonuse among women (15–49 years) and socio-economic and demographic factors across regions of the country.

## Methods

### Data used

**Fig. 1:**
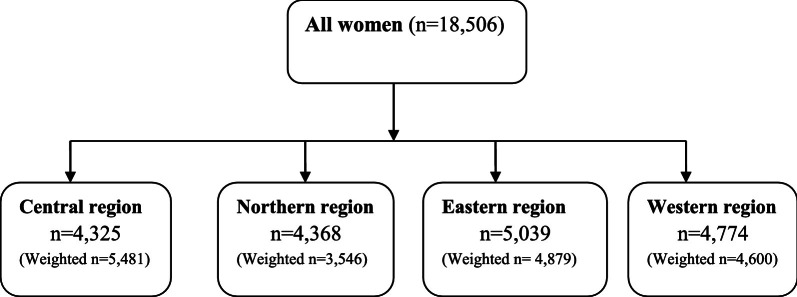
Origin of the sample adopted for the study

This paper used secondary data from the 2016 Uganda Demographic and Health Survey (UDHS). Details regarding sampling in the 2016 UDHS can be obtained elsewhere [10]. In the present study, we obtained access and permission to download and use the 2016 UDHS data from DHS program web platform, after submitting the study proposal. This study utilized the women’s questionnaire that focused on women of reproductive age (15–49 years). In the 2016 UDHS women’s questionnaire, women of reproductive age were asked about whether they have ever used anything or tried to delay or avoid getting pregnant; this was used as a measure of contraceptive nonuse in this study. This study incorporated all women aged 15–49 (18,506); these women are exposed to the risk of pregnancy [10]. Women utilize contraceptives in order to reduce on the risk of unwanted pregnancies and child birth [27–29]. This study regrouped the fifteen (15) regions in the 2016 UDHS into four (4) regions of Uganda for the rationale of analysis; Central (Kampala, south Buganda, and North Buganda), Eastern (Busoga, Bukedi, Bugisu, and Teso), Western (Bunyoro, Tooro, Ankole, and Kigezi), and Northern regions (Lango, Acholi, Karamoja, and West nile) [28, 30]. In order to ensure that the sampled data was representative, and adjusted for non-responses in the country, data was weighted and compound design in analyses while utilizing the SVY command in STATA 13.0 was considered. Figure 1 is a flow diagram that indicates the derivation of the study sample.

### Variables

The dependent variable of this study was contraceptive nonuse. This was assessed using a binary outcome; whether a woman has never used anything or tried to delay or avoid getting pregnant (coded 1), and or has ever used anything or tried to delay or avoid getting pregnant (coded 0). In this study, the independent variables used include; age of the respondent, respondent’s desire for children, respondent’s number of living children, number of children born to the respondent in the last 5 years, employment status of the respondent, respondent’s education level, respondent’s place of residence, religious affiliation of the respondents, household wealth index, age at first marriage, age at first sex, radio listening, television watching, reading newspaper or magazines, perception of distance to health facility, sexual autonomy and age at first birth. Women who reported a frequency of having sexual freedom and, listening/watching and reading newspapers or magazines were grouped as “yes” whereas women who reported no frequency were grouped as “no”.

### Statistical analysis

Analysis was done using STATA 13.0. Three stages were undertaken, which included; first, with the use of frequency distributions, we created descriptive summaries on women’s demographic and socio-economic characteristics across all the regions of Uganda. Second, analysis of variation in contraceptive nonuse by women’s demographic and socio-economic factors across all the regions of the country was done through a cross-tabular analysis with relationships investigated using Pearson Chi-square test. Third, net-association of women’s demographic and socio-economic characteristics on contraceptive nonuse was done with a logistic regression analysis to obtain the likelihood estimates of contraceptive nonuse among women across all the regions of Uganda. Logistic regression was adopted due to the nature of the modelled outcome variable (binary outcome). Odds ratios (OR’s) with 95% confidence interval were adopted in the presentation of study findings. Relationships with *p* values < 0.05 were considered statistical significant; additionally, *p* value < 0.001 indicated very strong relationships, *p* value < 0.01 showed a strong relationship, and *p* value < 0.05 indicated moderate relationships [31]. Archer-Lemeshow goodness of fit test was adopted in testing the suitability of the regression model using STATA 13.0 software [32].

## Results

### Descriptive findings of the respondents

Results (Table 1) indicate statistical significance of contraceptive nonuse across regions of the country. In the results, reveal the highest prevalence of contraceptive nonuse was in Northern region (55%), followed-by Western region (44%), and lowest in Central region (35%). Table 1 shows a distribution of contraceptive nonuse among women (15–49 years) across regions.

**Table 1:** Percentage distribution of contraceptives nonuse among women aged 15–49 years across regions

Fertility	Regions			
	Central (n = 4325)	Eastern (n = 5039)	Northern (n = 4368)	Western (n = 4774)
Contraceptive nonuse	1491 (35%)	2153 (43%)	2420 (55%)	2084 (44%)
Contraceptive use	2834 (65%)	2886 (57%)	1947 (45%)	2690 (56%)

χ^2^ = 386.6, *p* = 0.0000

Results in Table 2 indicate selected women’s socio-economic and demographic characteristics across regions. Most respondents have primary educational level, with the highest proportion in Northern region (67.1%). Also, Northern region (49.8%) unlike other regions had the highest proportion of women in the poorest wealth index. Across regions, more than half of the respondents reside in the rural areas. Additionally, the results also reveal that several respondents across regions married before their 18 birthday; with majority of respondents in Northern region (51%), followed-by Eastern region (48%), and lowest in Central region (37.9%). In addition, results depict that over 50% of the respondents across all regions perceived no problem with distance to health facility. Besides, majority of the respondents were unemployed, with highest proportion in Northern region (80.7%).

**Table 2:** Percentage distribution of women’s socio-economic and demographic factors across regions

Characteristics	Regions			
	Central (n = 4325)	Eastern (n = 5039)	Northern (n = 4368)	Western (n = 4600)
*Age*				
15–19	894.4 (20.7%)	1288.7 (25.6%)	1111.1 (25.4%)	1019.3 (21.4%)
20–24	993.9 (23.0%)	1014.4 (20.1%)	834.9 (19.1%)	936.5 (19.6%)
25–29	791.0 (18.3%)	741.3 (14.7%)	669.4 (15.3%)	817.7 (17.1%)
30–34	572.6 (13.2%)	652.0 (12.9%)	633.9 (14.5%)	697.0 (14.6%)
35–39	485.9 (11.2%)	505.5 (10.0%)	455.0 (10.4%)	556.9 (11.7%)
40–44	338.8 (7.8%)	489.8 (9.7%)	385.5 (8.8%)	406.0 (8.5%)
45–49	249.5 (5.8%)	347.5 (6.9%)	278.2 (6.4%)	340.7 (7.1%)
Total (%)	100	100	100	100
*Educational level*				
No education	231.3 (5.4%)	352.1 (7.0%)	751.1 (17.2%)	557.8 (11.7%)
Primary	1862.4 (43.1%)	3116.0 (61.8%)	2931.4 (67.1%)	2981.7 (62.5%)
Secondary	1613.3 (37.3%)	1293.2 (25.7%)	495.2 (11.3%)	975.9 (20.4%)
Higher	618.0 (14.3%)	277.7 (5.5%)	190.3 (4.4%)	258.7 (5.4%)
Total (%)	100	100	100	100
*Religion*				
Catholic	1511.0 (34.9%)	1403.9 (27.9%)	2587.1 (59.2%)	2034.7 (42.6%)
Anglican	1138.6 (26.3%)	1845.6 (36.6%)	991.0 (22.7%)	1805.5 (37.8%)
Muslim	794.2 (18.4%)	866.1 (17.2%)	409.8 (9.4%)	218.6 (4.6%)
Pentecostal	742.5 (17.2%)	799.1 (15.9%)	360.6 (8.3%	478.1 (10.0%)
Others	138.8 (3.2%)	124.3 (2.5%)	19.6 (0.5%)	237.1 (5.0%)
Total (%)	100	100	100	100
*Age at first marriage*				
≤ 12	90.4 (3.0%)	140.5 (3.7%)	119.6 (3.6%)	142.0 (3.9%)
13–17	1150.9 (37.9%)	1811.1 (48.0%)	1706.8 (51.0%)	1474.8 (40.8%)
18–24	1519.4 (50.1%)	1578.5 (41.8%)	1342.8 (40.1%)	1748.2 (48.4%)
25+	275.4 (9.1%)	242.9 (6.4%)	117.7 (5.3%)	247.0 (6.8%)
Total (%)	100	100	100	100
*Sexual autonomy*				
No	144.3 (6.2%)	493.5 (15.2%)	488.8 (17.6%)	457.5 (15.1%)
Yes	2171.8 (93.0%)	2722.9 (84.0%)	2275.2 (81.9%)	2489.5 (82.4%)
Don’t know	20.0 (0.9%)	24.7 (0.8%)	15.1 (0.5%)	74.0 (2.5%)
Total (%)	100	100	100	100
*Wealth index*				
Poorest	110.4 (2.6%)	982.4 (19.5%)	2174.5 (49.8%)	405.1 (8.5%)
Poorer	349.6 (8.1%)	1224.3 (24.3%)	964.5 (22.1%)	1022.6 (21.4%)
Middle	555.6 (12.9%)	1084.3 (21.5%)	469.6 (10.8%)	1374.8 (28.8%)
Richer	948.2 (21.9%)	1031.4 (20.5%)	431.7 (9.9%)	1175.2 (24.6%)
Richest	2361.2 (54.6%)	716.7 (14.2%)	327.8 (7.5%)	796.4 (16.7%)
Total (%)	100	100	100	100
*Residence*				
Urban	2075.1 (48.0%)	798.0 (15.8%)	654.7 (15.0%)	1047.5 (21.9%)
Rural	2249.9 (52.0%)	4241.0 (84.2%)	3713.3 (85.0%)	3726.5 (78.1%)
Total (%)	100	100	100	100
*Age at first sex*				
Not had sex	606.9 (14.1%)	715.3 (14.2%)	692.6 (15.9%)	697.5 (14.6%)
Below 15	578.6 (13.4%)	1065.5 (21.2%)	606.2 (13.9%)	767.5 (16.1%)
15–19	2591.6 (60.0%)	2966.6 (58.9%)	2695.4 (61.7%)	2749.2 (57.6%)
20–24	493.3 (11.4%)	270.1 (5.4%)	331.2 (7.6%)	504.1 (10.6%)
25+	49.5 (1.1%)	20.6 (0.4%)	41.6 (0.9%)	54.8 (1.1%)
Total (%)	100	100	100	100
*Age at first birth*				
≤ 14	231.4 (7.5%)	312.7 (8.4%)	221.3 (6.7%)	249.7 (6.9%)
15–19	1684.0 (54.3%)	2421.6 (64.7%)	2055.6 (62.6%)	1987.4 (54.9%)
20–24	967.2 (31.2%)	854.5 (22.8%)	865.2 (26.4%)	1135.3 (31.4%)
25+	219.4 (7.1%)	153.2 (4.1%)	138.9 (4.1%)	247.7 (6.8%)
Total (%)	100	100	100	100
*Perception of distance to health facility*				
A big problem	1118.1 (25.8%)	1990.5 (39.5%)	2162.2 (49.5%)	1884.2 (39.5%)
Not a big problem	3206.9 (74.2%)	3048.5 (60.5%)	2205 (50.5%)	2889.8 (60.5%)
Total (%)	100	100	100	100
*Desire for children*				
Wants within 2 years	632.1 (14.6%)	542.0 (10.8%)	477.0 (10.9%)	547.4 (11.5%)
Wants after 2 years	1655.5 (38.3%)	2049.3 (40.7%)	1953.7 (44.7%)	1810.5 (37.9%)
Wants, but unsure of timing	519.2 (12.0%)	465.3 (9.2%)	334.6 (7.7%)	445.1 (9.3%)
Undecided	179.4 (4.2%)	215.7 (4.3%)	165.6 (3.8%)	179.9 (3.8%)
Wants no more	1338.8 (31.0%)	1766.7 (35.1%)	1437.2 (32.9%)	1791.2 (37.5%)
Total (%)	100	100	100	100
*Number of living children*				
0	1244.5 (28.8%)	1.358.3(27.0%)	1140.1 (26.1%)	1172.1 (24.6%)
1	693.6 (16.0%)	615.0 (12.2%)	584.9 (13.4%)	667.7 (14.0%)
2	619.4 (14.3%)	609.3 (12.1%)	531.3 (12.2%)	653.7 (13.7%)
3+	1767.6 (40.9%)	2456.5 (48.8%)	2111.7 (48.3%)	2280.5 (47.8%)
Total (%)	100	100	100	100
*Number of children born in the 5 years*				
0	2111.7 (48.8%)	2222.3 (44.1%)	1829.7 (41.9%)	2117.7 (44.4%)
1	1328.7 (30.7%)	1442.3 (28.6%)	1464.5 (33.5%)	1523.3 (31.9%)
2+	884.6 (20.5%)	1374.4 (27.3%)	1073.9 (24.6%)	1133.0 (23.7%)
Total (%)	100	100	100	100
*Listen to radio*				
No	680.6 (15.7%)	1450.8 (28.8%)	1550.0 (35.5%)	1298.8 (27.2%)
Yes	3644.4 (84.3%)	3588.2 (71.2%)	2818.1 (64.5%)	3475.2 (72.8%)
Total (%)	100	100	100	100
*Watch television*				
No	1732.4 (40.1%)	3793.5 (75.3%)	3728.7 (85.4%)	3714.9 (77.8%)
Yes	2592.6 (59.9%)	1245.6 (24.7%)	639.3 (14.6%)	1059.1 (22.2%)
Total (%)	100	100	100	100
*Reading of newspaper or magazine*				
No	2536.9 (58.7%)	4034.1 (80.1%)	3890.5 (89.1%)	4038.2 (84.6%)
Yes	1788.1 (41.3%)	1005.0 (19.9%)	477.5 (10.9%)	735.8 (15.4%)
Total (%)	100	100	100	100
*Employment status*				
Unemployed	1310.1 (30.3%)	1413.6 (28.1%)	843.2 (19.3%)	1320.3 (27.7%)
Employed	3014.9 (69.7%)	3625.4 (71.9%)	3524.8 (80.7%)	3453.7 (72.3%)
Total (%)	100	100	100	100

Weighted percentage distributions

### Analysis of variations in respondent’s socio-economic and demographic predictors by contraceptive nonuse across regions

This study examined socio-economic and demographic predictors of contraceptive nonuse at bivariate level of analysis. At this level, associations were investigated using Pearson chi-square test that compared differences in contraceptive nonuse by socio-economic and demographic predictors across regions. In the results (Table 3), respondents’ age, age at first sex, number of children born in the last five (5) years, listening to radio and employment status was statistically significant across regions $\left( {p < 0.05} \right)$. Educational level, religious status, age at first marriage, desire for children, sexual autonomy, wealth quintile, residence, age at first birth, and watching television were only statistically significant in some regions. For perception of distance to the health facility and reading newspapers or magazines, these were not significant across regions. Therefore, due to the importance of all the variables in predicting contraceptive nonuse, they were adopted at the multivariate level of analysis.

**Table 3:** Percentage distribution of socio-economic and demographic characteristics of respondents by contraceptive nonuse across regions

Variables	Regions							
	Central		Eastern		Northern		Western	
	Non-use	Use	Non-use	Use	Non-use	Use	Non-use	Use
*Age*								
15–19	679.7 (76.1%)	213.7 (23.9%)	1021.1 (79.2%)	267.5 (20.8%)	971.9 (87.5%)	139.2 (12.5%)	872.2 (85.6%)	147.1 (14.4%)
20–24	329.4 (33.1%)	664.5 (66.9%)	392.1 (38.7%)	622.3 (61.3%)	441.0 (52.8%)	393.9 (47.2%)	388.8 (41.5%)	547.7 (58.5%)
25–29	135.8 (17.2%)	655.2 (82.8%)	161.9 (21.9%)	579.3 (78.1%)	242.6 (36.2%)	426.8 (63.8%)	223.5 (27.3%)	594.1 (72.7%)
30–34	94.4 (16.5%)	478.2 (83.5%)	139.8 (21.4%)	512.2 (78.6%)	222.3 (35.1%)	411.6 (64.9%)	164.1 (23.6%)	532.9 (76.4%)
35–39	96.6 (19.9%)	389.3 (80.1%)	130.1 (25.7%)	375.4 (74.3%)	179.3 (39.4%)	275.7 (60.6%)	150.4 (27.0%)	406.5 (73.0%)
40–44	77.6 (22.9%)	261.2 (77.1%)	151.6 (30.9%)	338.2 (69.1%)	184.0 (47.7%)	201.5 (52.3%)	146.6 (36.1%)	259.5 (63.9%)
45–49	77.7 (31.1%)	171.8 (68.9%)	156.1 (44.9%)	191.4 (55.1%)	179.7 (64.6%)	98.6 (35.4%)	138.6 (40.7%)	202.1 (59.3%)
	χ^2^ = 939.3498, *p* = 0.0000		χ^2^ = 1049.7960, *p* = 0.0000		χ^2^ = 736.1774, *p* = 0.0000		χ^2^ = 1006.2228, *p* = 0.0000	
*Educational level*								
No education	81.8 (35.4%)	149.5 (64.6%)	170.7 (48.5%)	181.4 (51.5%)	494.4 (65.8%)	256.7 (34.2%)	253 (45.4%)	304.8 (54.6%)
Primary	652.8 (35.1%)	1209.7 (64.9%)	1351.6 (43.4%)	1764.5 (56.6%)	1607.9 (54.9%)	1323.5 (45.1%)	1326 (44.5%)	1655.7 (55.5%)
Secondary	581.1 (36.0%)	1032.2 (64.0%)	556.5 (43.0%)	736.6 (57.0%)	254.3 (51.4%)	240.9 (48.6%)	482.6 (43.9%)	547.2 (56.1%)
Higher	175.6 (28.4%)	442.5 (71.6%)	73.9 (26.6%)	203.7 (73.4%)	64.1 (33.7%)	126.2 (66.3%)	76.6 (29.6%)	182.1 (70.4%)
	χ^2^ = 12.1249, *p* = 0.0995		χ^2^ = 34.7707, *p* = 0.0007		χ^2^ = 73.0577, *p* = 0.0000		χ^2^ = 22.2693, *p* = 0.0008	
*Religious status*								
Catholics	476.8 (31.6%)	1034.2 (68.4%)	623.4 (44.4%)	780.5 (55.6%)	1485 (57.4%)	1102.1 (42.6%)	907.3 (44.6%)	1127.5 (55.4%)
Anglican	379.2 (33.3%)	759.4 (66.7%)	794.2 (43.0%)	1051.4 (57.0%)	468.7 (47.3%)	522.2 (52.7%)	737.3 (40.8%)	1068.1 (59.2%)
Muslim	261.7 (32.9%)	532.5 (67.1%)	342.9 (39.6%)	532.3 (60.4%)	269.3 (65.7%)	140.5 (34.3%)	92.6 (42.4%)	126.0 (57.6%)
Pentecostal	313.1 (42.2%)	429.4 (57.8%)	353.8 (44.3%)	445.3 (55.7%)	183.1 (50.8%)	177.5 (49.2%)	220.1 (46.0%)	258.0 (54.0%)
Others	60.5 (43.6%)	78.3 (56.4%)	38.5 (31.0%)	85.8 (69.0%)	14.6 (74.4%)	5.0 (25.6%)	126.9 (53.5%)	110.2 (46.5%)
	χ^2^ = 31.7611, *p* = 0.0005		χ^2^ = 12.9586, *p* = 0.0870		χ^2^ = 54.1094, *p* = 0.0000		χ^2^ = 17.2144, *p* = 0.0204	
*Age at first marriage*								
≤ 12	17.9 (19.9%)	72.4 (80.1%)	50.4 (35.8%)	90.2 (64.2%)	49.8 (41.6%)	69.8 (58.4%)	63.0 (44.4%)	79.0 (55.6%)
13–17	231.0 (20.1%)	919.9 (79.9%)	555.2 (30.7%)	1255.9 (69.3%)	712.5 (41.7%)	994.3 (58.3%)	459.2 (31.1%)	1,015.7 (68.9%)
18–24	316.4 (20.8%)	1203 (79.2%)	460.7 (29.2%)	1117.8 (70.8%)	657.6 (49.0%)	685.3 (51.0%)	542.8 (31.0%)	1,205.4 (69.0%)
25+	316.4 (19.4%)	222.1 (80.6%)	78.3 (32.2%)	164.6 (67.8%)	84.4 (47.5%)	93.4 (52.5%)	79.7 (32.3%)	167.2 (67.7%)
	χ^2^ = 0.4413 *p* = 0.9561		χ^2^ = 3.5023 *p* = 0.4181		χ^2^ = 16.8430 *p* = 0.0028		χ^2^ = 11.1670 *p* = 0.0394	
*Sexual autonomy*								
No	44.9 (31.1%)	99.3 (68.9%)	175.7 (35.6%)	317.8 (64.4%)	227.9 (46.6%)	260.9 (53.4%)	168.7 (36.9%)	288.9 (63.1%)
Yes	405.9 (18.7%)	1765.8 (81.3%)	801.8 (29.5%)	1921.1 (70.5%)	1021.3 (44.9%)	1253.9 (55.1%)	738.6 (29.7%)	1750.9 (70.3%)
Don’t know	1.5 (7.7%)	18.5 (92.3%)	6.4 (26.0%)	18.2 (74.0%)	4.4 (29.4%)	10.6 (70.6%)	16.6 (22.5%)	57.3 (77.5%)
	χ^2^ = 15.2060, *p* = 0.0024		χ^2^ = 7.7185, *p* = 0.0724		χ^2^ = 1.9994, *p* = 0.4484		χ^2^ = 11.7622, *p* = 0.0070	
*Wealth quintile*								
Poorest	46.6 (42.2%)	63.8 (57.8%)	436.6 (44.4%)	545.8 (55.6%)	1348 (62.0%)	826.4 (38.0%)	246.7 (60.9%)	158.4 (39.1%)
Poorer	142.9 (40.9%)	206.7 (59.1%)	530.9 (43.4%)	693.4 (56.6%)	480.1 (49.8%)	484.5 (50.2%)	476.6 (46.9%)	543.0 (53.1%)
Middle	191.1 (34.4%)	364.5 (65.6%)	484.4 (44.7%)	599.9 (55.3%)	221.7 (47.2%)	247.9 (52.8%)	610.2 (44.4%)	764.5 (55.6%)
Richer	322.2 (34.0%)	626.0 (66.0%)	419.5 (40.7%)	612.0 (59.3%)	206.2 (47.8%)	225.4 (52.2%)	451.6 (38.4%)	723.5 (61.6%)
Richest	788.5 (33.4%)	1572.8 (66.6%)	281.5 (39.3%)	435.2 (60.7%)	164.7 (50.3%)	163.1 (49.7%)	296.0 (37.2%)	500.3 (62.8%)
	χ^2^ = 10.5514 *p* = 0.225		χ^2^ = 8.3496 *p* = 0.2145		χ^2^ = 77.0443 *p*.0.0000		χ^2^ = 80.3140 *p* = 0.0000	
*Residence*								
Urban	675.5 (32.5%)	1399.6 (67.5%)	302.4 (37.9%)	495.6 (62.1%)	359.3 (54.9%)	295.3 (45.1%)	389.1 (37.1%)	658.5 (62.9%)
Rural	815.7 (36.3%)	1434.2 (63.7%)	1850.4 (43.6%)	2390.6 (56.4%)	2061.4 (55.5%)	1651.9 (44.5%)	1695.2 (45.5%)	2031.3 (54.5%)
	χ^2^ = 6.5424 *p* = 0.1226		χ^2^ = 9.0415 *p* = 0.0170		χ^2^ = 0.0884 *p* = 0.8895		χ^2^ = 23.1874 *p* = 0.0080	
*Age at first sex*								
Not had sex	601.4 (99.1%)	5.6 (0.9%)	703.9 (98.4%)	11.4 (1.6%)	686.7 (99.2%)	5.9 (0.9%)	694.9 (99.6%)	2.5 (0.4%)
Below 15	134.8 (23.3%)	443.8 (76.7%)	341.5 (32.0%)	723.9 (68.0%)	276.2 (45.6%)	330.0 (54.4%)	287.3 (37.4%)	480.1 (62.6%)
15–19	597.5 (23.0%)	1994.2 (77.0%)	1009.4 (34.0%)	1957.2 (66.0%)	1258.3 (46.7%)	1437.1 (53.3%)	917.4 (33.4%)	1831.8 (66.6%)
20–24	136.1 (27.6%)	357.2 (72.4%)	85.0 (31.5%)	185.0 (68.5%)	167.8 (50.7%)	163.4 (49.3%)	160.7 (31.9%)	343.4 (68.1%)
25+	17.5 (35.3%)	32.0 (64.7%)	12.4 (60.3%)	8.2 (39.7%)	30.6 (73.5%)	11.0 (26.5%)	22.5 (41.1%)	32.3 (58.9%)
	χ^2^ = 1314.3009 *p* = 0.0000		χ^2^ = 1064.1139 *p* = 0.0000		χ^2^ = 651.7632 *p* = 0.0000		χ^2^ = 1047.4633 *p* = 0.0000	
*Age at first birth*								
Below 15	38.0 (16.4%)	193.4 (83.6%)	87.3 (27.9%)	225.4 (72.1%)	82.1 (37.1%)	139.2 (62.9%)	97.6 (39.1%)	152.1 (60.9%)
15–19	307.2 (18.2%)	1376.8 (81.8%)	698.5 (28.8%)	1723.2 (71.2%)	832.3 (40.5%)	1223.4 (59.5%)	536.2 (27.0%)	1451.2 (73.0%)
20–24	187.0 (19.3%)	780.3 (80.7%)	253.1 (29.6%)	601.4 (70.4%)	431.5 (49.9%)	433.6 (50.1%)	350.7 (30.9%)	784.7 (69.1%)
25+	56.5 (25.8%)	162.8 (74.2%)	55.2 (36.0%)	98.0 (64.0%)	72.9 (52.5%)	66.0 (47.5%)	100.2 (40.5%)	147.4 (59.5%)
	χ^2^ = 8.2373 *p* = 0.1196		χ^2^ = 3.9420 *p* = 0.3506		χ^2^ = 30.1347 *p* = 0.0000		χ^2^ = 31.8334 *p* = 0.0001	
*Perception of distance to health facility*								
A big problem	360.3 (32.2%)	757.8 (67.8%)	861.4 (43.3%)	1129.1 (56.7%)	1163.2 (53.8%)	999.0 (46.2%)	845.0 (44.9%)	1039.1 (55.1%)
Not a big problem	1130.9 (35.3%)	2076.0 (64.7%)	1291.4 (42.4%)	1757.1 (57.6%)	1257.5 (57.0%)	948.3 (43.0%)	1239.2 (42.9%)	1650.6 (57.1%)
	χ^2^ = 3.3924 *p* = 0.1179		χ^2^ = 0.4099 *p* = 0.5777		χ^2^ = 4.5650 *p* = 0.1119		χ^2^ = 1.7954 *p* = 0.2951	
*Desire for children*								
Wants within 2 years	199.0 (31.5%)	433.1 (68.5%)	200.4 (37.0%)	341.6 (63.0%)	289.9 (60.8%)	187.1 (39.2%)	274.4 (50.1%)	273.0 (49.9%)
Wants after 2 years	571.1 (34.5%)	1084.5 (65.5%)	941.3 (45.9%)	1107.9 (54.1%)	1092.1 (55.9%)	861.6 (44.1%)	847.3 (46.8%)	963.2 (53.2%)
Wants, but unsure of timing	348.2 (67.1%)	170.9 (32.9%)	351.6 (74.6%)	113.8 (24.4%)	303.0 (90.5%)	31.7 (9.5%)	351.3 (78.9%)	93.8 (21.1%)
Undecided	85.3 (47.6%)	94.1 (52.4%)	131.4 (60.9%)	84.3 (39.1%)	102.5 (61.9%)	63.0 (38.1%)	80.1 (44.5%)	99.8 (55.5%)
Wants no more	287.6 (21.5%)	1051.2 (78.5%)	528.1 (29.9%)	1238.6 (70.1%)	633.2 (44.1%)	803.9 (55.9%)	531.2 (29.7%)	1260 (70.3%)
	χ^2^ = 360.4156 *p* = 0.0000		χ^2^ = 368.8890 *p* = 0.0000		χ^2^ = 250.5904 *p* = 0.0000		χ^2^ = 384.5077 *p* = 0.0000	
*Number of living children*								
0	919.4 (73.9%)	325.1 (26.1%)	1085.6 (79.9%)	272.7 (20.1%)	1037.8 (91.0%)	102.3 (9.0%)	1015.7 (86.7%)	156.4 (13.3%)
1	195.4 (28.1%)	498.1 (71.8%)	281.9 (45.8%)	333.1 (54.2%)	337.2 (57.7%)	247.6 (42.3%)	293.6 (44.0%)	374.1 (56.0%)
2	97.3 (15.7%)	522.1 (84.3%)	162.7 (26.7%)	446.6 (73.3%)	221.1 (41.6%)	310.3 (58.4%)	199.8 (30.6%)	454.0 (69.4%)
3+	279.1 (15.8%)	1488.6 (84.2%)	622.7 (25.4%)	1834 (74.6%)	824.6 (39.1%)	1287.1 (60.9%)	575.2 (25.2%)	1705.3 (74.8%)
	χ^2^ = 1237.2615 *p* = 0.0000		χ^2^ = 1137.4900 *p* = 0.0000		χ^2^ = 856.3612 *p* = 0.0000		χ^2^ = 1241.7939 *p* = 0.0000	
*Number of children born in the last 5 years*								
0	1104.7 (52.3%)	1007.0 (47.7%)	1351.0 (60.8%)	871.4 (39.2%)	1364.3 (74.6%)	465.3 (25.4%)	1306.7 (61.7%)	811.0 (38.3%)
1	218.0 (16.4%)	1110.8 (83.6%)	391.8 (27.2%)	1050.6 (72.8%)	568.5 (38.8%)	896.0 (61.2%)	388.0 (25.5%)	1135.3 (74.5%)
2+	168.6 (19.1%)	716.0 (80.9%)	410.1 (29.8%)	964.3 (70.2%)	487.9 (45.4%)	585.9 (54.6%)	389.5 (34.4%)	743.5 (65.6%)
	χ^2^ = 582.6405 *p* ≤ 0.001		χ^2^ = 532.4315 *p* ≤ 0.001		χ^2^ = 478.2326 *p* ≤ 0.001		χ^2^ = 524.9012 *p* ≤ 0.001	
*Listen to radio*								
No	281.0 (41.3%)	399.6 (58.7%)	688.4 (47.5%)	762.4 (52.5%)	927.0 (59.8%)	623.0 (40.2%)	732.1 (56.4%)	566.7(43.6%)
Yes	1210.2 (33.2%)	2434.2 (66.8%)	1464.4 (40.8%)	2123.8 (59.2%)	1493.8 (53.0%)	1324.3 (47.0%)	1352.1 (38.9%)	2123.0 (61.1%)
	χ^2^ = 16.6029 *p* = 0.0020		χ^2^ = 18.6168 *p* = 0.0001		χ^2^ = 18.7018 *p* = 0.0021		χ^2^ = 117.1477 *p* = 0.0000	
*Watch television*								
No	634.1 (36.6%)	1098.3 (63.4%)	1640.6 (43.3%)	2152.9 (56.7%)	2071.9 (55.6%)	1656.8 (44.4%)	1663.9 (44.8%)	2051.0 (55.2%)
Yes	857.1 (33.1%)	1735.5 (66.9%)	512.2 (41.1%)	733.4 (58.9%)	348.8 (54.6%)	290.5 (45.5%)	420.3 (39.7%)	638.8 (60.3%)
	χ^2^ = 5.7687 *p* = 0.0621		χ^2^ = 1.7329 *p* = 0.2971		χ^2^ = 0.2241 *p* = 0.6942		χ^2^ = 8.7283 *p* = 0.0346	
*Reading of newspaper or magazine*								
No	880.2 (34.7%)	1656.7 (65.3%)	1737.3 (43.1%)	2296.8 (56.9%)	2166.5 (55.7%)	1724.0 (44.3%)	1773.7 (43.9%)	2264.6 (56.1%)
Yes	611.0 (34.2%)	1177.1 (65.8%)	415.5 (41.4%)	589.4 (58.6%)	254.2 (53.2%)	223.3 (46.8%)	310.6 (42.2%)	425.2 (57.8%)
	χ^2^ = 0.1270 *p* = 0.8192		χ^2^ = 0.9696 *p* = 0.4593		χ^2^ = 1.0307 *p* = 0.4541		χ^2^ = 0.7420 *p* = 0.4803	
*Employment status*								
Unemployed	611.3 (46.7%)	698.8 (53.3%)	847.4 (60.0%)	566.2 (40.0%)	630.6 (74.8%)	212.5 (25.2%)	842.1 (63.8%)	478.3 (36.2%)
Employed	879.9 (29.2%)	2135 (70.8%)	1305.4 (36.0%)	2320.1 (64.0%)	1790.1 (50.8%)	1734.7 (49.2%)	1242.2 (36.0%)	2211.5 (64.0%)
	χ^2^ = 123.4241 *p* = 0.0000		χ^2^ = 238.2390 *p* = 0.0000		χ^2^ = 158.7397 *p* = 0.0000		χ^2^ = 300.3493 *p* = 0.0000	

Non-use—never used or tried using a contraceptive

Use—Ever used or tried using a contraceptive

### Multivariate results

Table 4 shows results of a binary multivariable logistic regression on contraceptive nonuse by socio-economic and demographic predictors across regions. The results reveal that women’s educational level, wealth index, number of living children and number of children born in the last 5 years were the only significant predictors of contraceptive nonuse across regions $(p < 0.05)$. The results also demonstrate that respondents’ current age, religious affiliation, age at first marriage, respondent’s sexual autonomy, age at first sex, age at first birth, desire for children, listening to radio and employment status were only significant predictors of contraceptive nonuse in particular regions. Conversely, residence, perception of distance to health facility, watching television, and reading newspapers or magazines were not predictors of contraceptive nonuse in any of the regions $(p > 0.05)$.

**Table 4:** Results from a multivariate logistic regression on contraceptive nonuse and women’s demographic and socio-economic factors across all regions (UDHS 2016)

Variables	Regions							
	Central		Eastern		Northern		Western	
	OR	95% CI	OR	95% CI	OR	95% CI	OR	95% CI
*Age*								
15–19^a^	1.00		1.00		1.00		1.00	
20–24	0.74	0.36–1.51	0.71	0.47–1.07	0.70	0.44–1.12	0.56*	0.32–0.96
25–29	0.58	0.25–1.28	0.56*	0.33–0.94	0.68	0.39–1.16	0.60	0.33–1.09
30–34	0.64	0.26–1.55	0.68	0.39–1.18	0.66	0.37–1.18	0.69	0.36–1.31
35–39	1.39	0.55–3.55	0.89	0.48–1.62	0.80	0.43–1.49	1.10	0.55–2.20
40–44	1.10	0.39–3.08	1.13	0.59–2.12	1.15	0.58–2.24	2.01	0.97–4.16
45–49	2.29	0.77–6.79	1.99	0.96–4.11	2.13*	11.00–4.54	2.48*	1.12–5.48
*Educational level*								
No education^a^	1.00		1.00		1.00		1.00	
Primary	0.52**	0.32–0.84	0.44***	0.32–0.61	0.45***	0.35–0.57	0.67**	0.51–0.88
Secondary	0.28***	0.15–0.49	0.34***	0.21–0.51	0.36***	0.23–0.55	0.46***	0.31–0.69
Higher	0.28***	0.13–0.62	0.15***	0.07–0.33	0.20***	0.16–0.56	0.58	0.33–1.12
*Religion*								
Catholics^a^	1.00		1.00		1.00		1.00	
Anglican	0.88	0.61–1.27	1.09	0.86–1.38	0.56***	0.44–0.71	0.75*	0.60–0.94
Muslim	0.88	0.58–1.34	1.04	0.76–1.42	1.70**	1.19–2.41	0.77	0.46–1.28
Pentecostal	1.06	0.70–1.62	1.08	0.81–1.47	0.69*	0.49–0.97	1.16	0.84–1.61
Others	1.46	0.63–3.39	0.47*	0.24–0.94	1.21	0.43–3.38	1.57*	1.05–2.34
*Age at first marriage*								
≤ 12^a^	1.00		1.00		1.00		1.00	
13–17	0.76	0.32–1.78	0.72	0.38–1.34	1.43	0.79–2.60	0.62	0.36–1.07
18–24	0.81	0.33–1.96	0.79	0.41–1.52	1.64	0.89–3.03	0.55*	0.31–0.98
25 +	0.46	0.16–1.34	0.88	0.42–1.84	1.35	0.64–2.85	0.48	0.23–1.02
*Sexual autonomy*								
No^a^	1.00		1.00		1.00		1.00	
Yes	0.52**	0.31–0.85	0.87	0.68–1.11	1.09	0.85–1.39	0.80	0.62–1.04
Don’t know	0.24	0.03–1.64	0.74	0.21–2.66	0.44	0.11–1.84	0.51	0.23–1.12
*Wealth index*								
Poorest^a^	1.00		1.00		1.00		1.00	
Poorer	0.65	0.28–1.27	0.94	0.73–1.21	0.51***	0.40–0.65	0.55**	0.37–0.80
Middle	0.34**	0.15–0.68	0.91	0.68–1.22	0.33***	0.23–0.47	0.47***	0.32–0.69
Richer	0.42*	0.19–0.81	0.72*	0.52–0.99	0.37***	0.25–0.54	0.37***	0.25–0.56
Richest	0.50	0.20–0.97	0.58*	0.35–0.96	0.42**	0.24–0.72	0.27***	0.16–0.46
*Residence*								
Urban^a^	1.00		1.00		1.00		1.00	
Rural	1.38	0.94–2.04	1.40	0.97–2.03	0.99	0.72–1.39	1.34	0.99–1.82
*Age at first sex*								
Below 15^a^	1.00		1.00		1.00		1.00	
15–19	1.05	0.66–1.66	0.12	0.87–1.46	0.92	0.70–1.22	0.74*	0.55–0.98
20–24	1.40	0.70–2.79	1.79	0.46–1.98	0.73	0.47–1.15	0.76	0.49–1.16
25 +	4.01*	1.20–13.40	4.36	0.87–21.73	2.54	0.67–9.60	0.46	0.17–1.22
*Age at first birth*								
Below 15^a^	1.00		1.00		1.00		1.00	
15–19	1.45	0.66–3.20	0.97	0.62–1.52	0.87	0.57–1.34	0.71	0.44–1.16
20–24	2.29	0.94–5.57	1.18	0.70–1.98	1.31	0.81–2.13	0.99	0.58–1.73
25+	3.58*	1.28–10.03	1.35	0.61–3.01	1.16	0.53–2.52	1.25	0.62–2.53
*Perception of distance to health facility*								
A big problem^a^	1.00		1.00		1.00		1.00	
Not a big problem	0.88	0.64–1.22	0.94	0.77–1.14	1.08	0.89–1.31	1.01	0.83–1.24
*Desire for children*								
Wants within 2 years^a^	1.00		1.00		1.00		1.00	
Wants after 2 years	1.29	0.84–1.99	0.87	0.63–1.19	0.78	0.56–1.07	0.54***	0.39–0.74
Wants, but unsure of timing	1.47	0.57–3.78	1.89	0.89–3.98	0.81	0.25–2.65	0.79	0.35–1.75
Undecided	1.59	0.62–4.11	1.87*	1.05–3.34	0.59	0.30–1.18	0.97	0.57–1.67
Wants no more	0.78	0.45–1.35	0.77	0.54–1.11	0.74	0.52–1.05	0.38***	0.27–0.54
*Number of living children*								
0^a^	1.00		1.00		1.00		1.00	
1	0.15**	0.43–0.49	1.02	0.41–2.46	0.44	0.14–1.35	1.12	0.44–2.86
2	0.04***	0.01–0.14	0.36*	0.22–1.37	0.17**	0.06–0.53	0.39	0.15–1.02
3 +	0.03***	0.00–0.11	0.24**	0.15–0.96	0.10***	0.03–0.32	0.20**	0.08–0.55
*Number of children born in the last 5 years*								
0	1.00		1.00		1.00		1.00	
1	0.53**	0.33–0.84	0.77	0.56–1.07	0.68*	0.50–0.92	0.71*	0.52–0.96
2 +	1.26	0.77–2.06	1.64**	1.17–2.29	1.25	0.88–1.78	2.32***	1.65–3.25
*Listen to radio*								
No^a^	1.00		1.00		1.00		1.00	
Yes	0.76	0.52–1.13	0.90	0.73–1.12	0.95	0.78–1.17	0.63***	0.50–0.80
*Watch television*								
No^a^	1.00		1.00		1.00		1.00	
Yes	0.75	0.51–1.09	1.18	0.87–1.58	1.17	0.85–1.61	0.81	0.60–1.10
*Reading of newspaper or magazine*								
No^a^	1.00		1.00		1.00		1.00	
Yes	0.74	0.51–1.08	1.00	0.73–1.37	0.74	0.50–1.12	0.82	0.56–1.19
*Employment status*								
Unemployed^a^	1.00		1.00		1.00		1.00	
Employed	1.04	0.73–1.47	0.78*	0.61–0.99	0.88	0.67–1.15	0.75*	0.58–0.96

*OR* odds Ratio, *CI* Confidence interval

^*^p value < 0.05(moderate), **p value < 0.01 (strong), ***p value < 0.001 (very strong)

^a^Reference category

Educational level unevenly predicted contraceptive nonuse across regions. In the results, only secondary educational level compared to no educational level was a very strong predictor of contraceptive nonuse across all regions $(p < 0.001)$. Increased educational level was associated with lower odds of contraceptive nonuse across regions in comparison with no educational level. In addition, wealth index erratically predicted contraceptive nonuse across regions, although with lower odds of contraceptive nonuse among the poorer, middle, richer and richest women compared with the women in the poorest wealth index; for example, in Central region, the association between contraceptive nonuse and women in the middle and richer wealth index was strong and moderate, respectively unlike in Eastern region where in relationship was moderate.

Results also reveal that there were variations in effect between respondents’ number of children born in the last five (5) years and contraceptive nonuse across regions. Central region had a strong association and lower odds of contraceptive nonuse among women who had one child born in the last 5 years prior to the survey compared to women who never had a child born in the last 5 years prior the demographic survey $(p < 0.01)$. Unlike Central region, Northern and Western region had a moderate association with lower odds of contraceptive nonuse among women with one birth in the last 5 years prior to the survey, compared to the women with no birth at all in the last 5 years prior the survey $(p < 0.01)$.

Religion only predicted contraceptive nonuse in Eastern, Northern and Western region with variations. In Eastern region, religious category of “others” had moderate association and reduced odds of contraceptive nonuse compared with the “Catholics”. Also, women in the “Anglican” religion had very association with lower odds of contraceptive nonuse compared with their counterparts the “Catholics” $(p < 0.001)$. In addition, results showed that the “Muslim” in Northern region had a strong effect with increased odds of contraceptive nonuse compared with the “Catholics” $(p < 0.01)$. And, in the same region, “Pentecostal” with moderate association and lower odds of contraceptive nonuse compared with the “Catholics”$(p < 0.05)$.

Besides, results showed that age at first sex was not a determinant of contraceptive nonuse across regions, but a moderate $(p < 0.05)$ determinant of contraceptive nonuse in Central and Western region. In Central region, women aged 25 and above were four times more likely not to use contraceptives compared with the women whose age at first sex was below 15 years. In Western region, instead women whose age at first sex was 15–19 had reduced odds of contraceptive nonuse compared to the women whose age at first sex was below the age 15.

## Discussion

The study provided a wider assessment of socio-economic and demographic determinants of contraceptive nonuse across regions in Uganda among women aged 15–49 using the 2016 Uganda Demographic and Health Survey data. Again, amidst differences in effect, the study found educational level, wealth index number of living children and numbers of children born in the last 5 years prior to the demographic survey as the only predictors of contraceptive nonuse across all regions. Respondents’ age, religion, age at first marriage, age at first birth, Sexual autonomy, age at first sex, desire for children, listen to radio, and employment status were predictors of contraceptive nonuse in particular regions.

No study in Uganda can justify the differences in effect of women’s educational level on contraceptive nonuse across all regions. Although, studies done in Uganda and elsewhere generally associate less schooling of women to contraceptive nonuse [28, 33–37]; linked to lack of knowledge, fatalism, and lack of contraceptive access [34, 38]. Nevertheless, promotion of women’s educational advancements to at least secondary level across regions should be advanced because this could expedite on contraceptive utilization across all regions in Uganda. However, more research needs to be directed towards understanding these variations in effect of the same variably on contraceptive nonuse across regions.

Wealth index variably predicted contraceptive nonuse across regions as explained above. However, no study can clarify these variations in impact of wealth index on women’s contraceptive nonuse across all regions. Although, numerous studies have indicated that women in higher wealth index are associated with reduced odds of contraceptive nonuse compared to their counterparts the poorest women [2, 33, 37]. This kind of behavioural pattern has been associated to poverty among women in developing countries [38, 39]. Therefore, there is need to advance research towards understanding these variations in effect of wealth index on contraceptive nonuse across all regions.

Further, respondents’ number of living children differently predicted contraceptive nonuse across regions as earlier noted. Remarkably, no study in Uganda explains this result; although, studies elsewhere indicate that women with one, or more children have a higher chance of limiting child bearing compared to the women with no children who want to have a child [10, 40–44]. However, more research should be directed towards understanding the effect of respondents’ number of living children on contraceptive nonuse in the four regions.

Besides that, there have also not been studies that justify the variance in effect in association of respondents’ births in the last 5 years prior to the demographic health survey on contraceptive nonuse across regions of Uganda. Although, a related study among young women in Uganda revealed that women who had a birth in the last 5 years prior to the survey were five times more likely to use contraceptives compared to those who had never had a birth [45]. Nonetheless, extensive studies ought to be done to comprehensively understand the study finding across regions.

Contraceptive nonuse varied by women’s age in particularly Eastern Northern and Western region. Somewhat related with the study finding, a non-disintegrated study by region in Uganda revealed that contraceptive use among young women was low [45]; which could be associated to cost, fear, and cultural barriers [46, 47]. In addition, studies indicate that women aged 45–49 older aged are generally expected to have achieved their desired number of children; therefore, are associated with infrequent sexual intercourse, menopause, and sometimes lost interest for sex [48, 49]. Similarly, it is believed that women who are above the age of 25 have attained their fertility desires and therefore more likely to use contraceptives [48]. On the contrary, a study revealed that women aged 35–39 were more likely than women aged 25–29 to use contraceptives [50]. Conversely, investigations should be directed towards understanding extensively the findings of the study.

Religion differently predicted contraceptive nonuse in particularly Eastern, Northern and Western region. Importantly, there is scarce literature to justify the study findings. However, studies elsewhere indicate that lower odds of contraceptive nonuse among the Anglican and Pentecostal religion could be attributed to the absence of restrictions in contraceptive use compared to the Catholic religion that prohibits the use of contraceptives [51, 52]. Further, results show that Muslim women in Northern region had greater odds of contraceptive nonuse compared to the Catholics; this study finding contradicts with studies that reveal permissiveness of Islam religion in matters of contraceptive use [53, 54]. Conversely, studies into the variations in impact of religion on contraceptive nonuse should be done in the specific regions.

Age at first marriage predicted contraceptive nonuse in only Western region. In regard to the finding, there has not been any study that can explain this result. Although contrary to the finding, a related study in Uganda did not find any association between age at first marriage and contraceptive use [28]. In support of the result, a study found that older age at first marriage was linked to contraceptive nonuse compared to young age at first marriage [41]. This could be because young ages are usually associated with vulnerability that could easily culminate into contraceptive nonuse [55, 56]. There is therefore need to research into the association between contraceptive nonuse and age at first marriage of 18–24 in Western region.

Sexual autonomy predicted contraceptive nonuse in only Central region. There are no studies that justify this particular effect of sexual autonomy on contraceptive nonuse. However, the outcome could be attributed to empowerment, as this makes them have liberty over their bodies and even make knowledgeable choices pertaining contraception uptake [35, 57–60]. Therefore, to comprehensively understand this effect in Central region, advanced research should be done.

Furthermore, results revealed that age at first sex predicted contraceptive nonuse in only Central and Western region. Conversely, there has not been any study to justify this result. The findings in particularly Central and Western region demonstrate that this outcome could be associated with them being in stable relationships unlike their counterparts younger ages [61, 62].

Besides that, the desire for children by respondents’ differently predicted contraceptive nonuse in particularly Eastern and Western region. Notably, no study has been undertaken to provide an explanation for this result. Nevertheless, the study finding in Western region resonates with numerous studies [33, 42, 63]. In addition, results in Eastern region are in agreement with a study done by Ahmed Zohirul Islam [64]. However, studies towards holistically understanding the study findings need to be undertaken in the regions.

Listening to radio predicted contraceptive nonuse among women in only Western region. This study could not overtly associate listenership to radio among women in the region. Although, studies non-separated by region suggest that radio is a passage of contraceptive messages and therefore influences contraceptive nonuse [65–67]. However, inquiries should be directed towards understanding the study finding in Western region.

Employment status was a predictor of contraceptive nonuse in Eastern and Western region. Remarkably, no study has been done to explain this study finding. However, non-disintegrated studies suggest that employed women have reduced odds of contraceptive nonuse compared to the unemployed women [25, 33, 42]. This has been linked to the ability to control and make autonomous decisions [25, 68, 69]. Conversely, investigations should be undertaken to understand the impact of employment on contraceptive nonuse in the specific regions.

Nevertheless, this study was limited due to impossibility of determining direction of connectedness of relationships between contraceptive nonuse and socio-economic and demographic variables due to the cross-sectional nature of the data.

## Conclusions

This study has identified four major predictors of contraceptive nonuse across all the regions of Uganda among women aged 15–49. Foremost, educational level, number of living children, wealth index and children born in the last 5 years prior to the survey erratically predicted contraceptive nonuse across all the regions. For particular regions; age, religion, age at first marriage, Sexual autonomy, age at first sex, age at first birth, desire for children, listening to radio, and employment status were predictors of contraceptive nonuse in particular regions of the country. However, residence, perception of distance to health facility, watching television, and reading newspapers or magazines did not predict contraceptive nonuse at all.

Therefore, for Uganda to address the challenges of contraceptive nonuse; deliberate efforts by government and stakeholders need to focus on understanding regional differences and effects of the factors that are associated with contraceptive nonuse. To this end, girl child education should be strengthened to enable completion of higher educational level. For particular regions, this study highlights the need by government and stakeholders to advance academic research in understanding the effect of these predictors on contraceptive nonuse. For example, to understand factors associated with the variations in effect of religion, desire for children, wealth index, age, age at first sex and number of living children on contraceptive nonuse across the regions. This study as well points at the need for researchers to understand the associations and magnitude between contraceptive nonuse and employment status in Eastern and Western region; contraceptive nonuse and radio listening in Western region; contraceptive nonuse and age at first sex in Central and Western region; contraceptive nonuse and age at first birth in Central region; contraceptive nonuse and age at first marriage in Western region; and non-se of contraceptives and the ability to refuse sex in Central region.

## Data Availability

DHS data is available to the public domain through Measure DHS website: https://dhsprogram.com/data/available-datasets.cfm.
